# Effect of the 2010 task force criteria on reclassification of cardiovascular magnetic resonance criteria for arrhythmogenic right ventricular cardiomyopathy

**DOI:** 10.1186/1532-429X-16-47

**Published:** 2014-07-04

**Authors:** Ting Liu, Amit Pursnani, Umesh C Sharma, Yongkasem Vorasettakarnkij, Daniel Verdini, Peerawut Deeprasertkul, Ashley M Lee, Heidi Lumish, Manavjot S Sidhu, Hector Medina, Stephan Danik, Suhny Abbara, Godtfred Holmvang, Udo Hoffmann, Brian B Ghoshhajra

**Affiliations:** 1 Department of Radiology, The First Affiliated Hospital of China Medical University, Shenyang, China; 2 Cardiac CT/MRI/PET Program, Department of Radiology, Massachusetts General Hospital, Boston, MA, USA; 3 Electrophysiology Laboratory, Mount Sinai St Luke's-Roosevelt Hospital, New York, NY, USA; 4 Cardiothoracic Imaging Division, Department of Radiology, UTSW Medical Center, Dallas, TX, USA

**Keywords:** Arrhythmogenic right ventricular cardiomyopathy, 2010 task force criteria, Cardiovascular magnetic resonance

## Abstract

**Background:**

We sought to evaluate the effect of application of the revised 2010 Task Force Criteria (TFC) on the prevalence of major and minor Cardiovascular Magnetic Resonance (CMR) criteria for Arrhythmogenic Right Ventricular Cardiomyopathy (ARVC) versus application of the original 1994 TFC. We also assessed the utility of MRI to identify alternative diagnoses for patients referred for ARVC evaluation.

**Methods:**

968 consecutive patients referred to our institution for CMR with clinical suspicion of ARVC from 1995 to 2010, were evaluated for the presence of major and minor CMR criteria per the 1994 and 2010 ARVC TFC. CMR criteria included right ventricle (RV) dilatation, reduced RV ejection fraction, RV aneurysm, or regional RV wall motion abnormalities. When quantitative measures of RV size and function were not available, and in whom abnormal size or function was reported, a repeat quantitative analysis by 2 qualified CMR physicians in consensus.

**Results:**

Of 968 patients, 220 (22.7%) fulfilled either a major or a minor 1994 TFC, and 25 (2.6%) fulfilled any of the 2010 TFC criterion. Among patients meeting any 1994 criteria, only 25 (11.4%) met at least one 2010 criterion. All patients who fulfilled a 2010 criteria also satisfied at least one 1994 criterion. Per the 2010 TFC, 21 (2.2%) patients met major criteria and 4 (0.4%) patients fulfilled at least one minor criterion. Eight patients meeting 1994 minor criteria were reclassified as satisfying 2010 major criteria, while 4 patients fulfilling 1994 major criteria were reclassified to only minor or no criteria under the 2010 TFC.

Eighty-nine (9.2%) patients had alternative cardiac diagnoses, including 43 (4.4%) with clinically significant potential ARVC mimics. These included cardiac sarcoidosis, RV volume overload conditions, and other cardiomyopathies.

**Conclusions:**

Application of the 2010 TFC resulted in reduction of total patients meeting any diagnostic CMR criteria for ARVC from 22.7% to 2.6% versus the 1994 TFC. CMR identified alternative cardiac diagnoses in 9.2% of patients, and 4.4% of the diagnoses were potential mimics of ARVC.

## Background

Arrhythmogenic Right Ventricular Cardiomyopathy (ARVC) is a rare autosomal dominant disorder that frequently manifests as ventricular tachycardia or sudden cardiac death in young patients. Histopathologically, it is characterized by progressive fibro-fatty replacement of the myocardium, leading to right ventricular dysfunction [1-5] and ventricular ectopy. Early diagnosis of ARVC is considered imperative for prognosis and management, which usually consists of implantation of an automatic implantable defibrillator. Definitive diagnosis of ARVC remains challenging due to a low disease prevalence and the lack of a single conclusive diagnostic test [6]. Per the 1994 task force criteria (TFC) of the European Society of Cardiology and of the Scientific Council on Cardiomyopathies of the International Society and Federation of Cardiology, a clinical diagnosis of ARVC is established on the basis of structural and functional alteration, electrocardiographic abnormalities, and a positive family history [7].

Cardiovascular magnetic resonance (CMR) has shown promise in the evaluation of patients with suspected ARVC, given its ability to provide highly accurate anatomical and functional information [8-14]. In 2010, TFC formally incorporated CMR findings (such as the presence of regional wall motion abnormalities and quantitative functional assessment) into the diagnostic criteria for ARVC [15]. The presence of fatty myocardial infiltration by CMR, which was initially thought to be important in the diagnosis of ARVC due to its unique pathological process, is included in neither the 1994 nor 2010 TFC due to its high prevalence in the normal aging patient population [16-18]. Vermes *et al*. have found that the 2010 TFC resulted in a higher specificity but a lower sensitivity in identifying patients with ARVC, compared to the 1994 TFC [19]. However, the utility of the 2010 imaging criteria has not been validated in large cohorts.

In this study, we sought to evaluate the effect of revised 2010 Task Force Criteria (TFC) on the prevalence of major and minor CMR criteria for ARVC versus the 1994 TFC with and without the presence of fatty infiltrate and abnormal Late Gadolinium Enhancement (LGE) as an additional criterion. In addition, we assessed the utility of CMR in identifying alternative diagnoses for patients referred for ARVC evaluation.

## Methods

Our institutional IRB granted a waiver for this retrospective research. HIPAA compliance was maintained throughout the study. No outside funding was received, and the authors maintained full control over the data.

### Study population

This retrospective cohort study consisted of 968 consecutive patients who were referred to our institution for CMR with clinical suspicion of ARVC between 1995 and 2010. Baseline characteristics including age, gender, height, and weight were extracted from clinical CMR reports and electronic medical records.

### CMR protocol

All CMR exams were performed on a 1.5 Tesla field strength scanner (Signa Excite HDXt platform, General Electric Healthcare, Milwaukee, USA) using a dedicated 8-channel cardiac coil with technical parameters recommended by the manufacturer and optimized on a case-by-case basis via direct physician supervision. After localizing images were obtained, high-resolution T1-weighted spin-echo images were acquired in an axial plane through the ventricles, followed by corresponding fat-suppressed T1 spin-echo images. Double inversion recovery fast spin echo T1 images were acquired with and without fat saturation in the short axis plane. Cine balanced-steady state free precession (SSFP) cine images were obtained in short axis, and two-, three- and four-chamber planes. Late Gadolinium Enhancement (LGE) images were obtained in the short axis view after intravenous administration of 0.2 mmol/kg Magnevist (gadopentetate dimeglumine, Bayer Healthcare Pharmaceuticals, Germany) with a preparatory null time selected for optimal nulling of normal myocardium. All quantitative parameters were measured using balanced-SSFP cine sequences (short axis view).

### CMR image analysis and TFC

**Figure 1:**
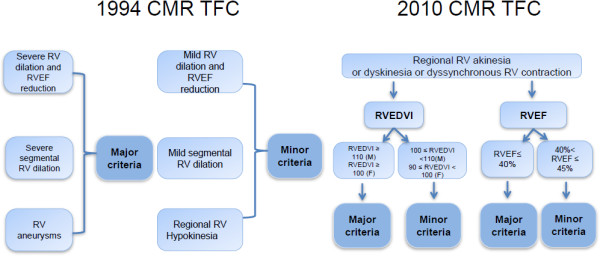
**1994 and 2010 ARVC CMR task force criteria.** The flow diagram shows comparison of the details for 1994 and 2010 ARVC CMR criteria.

The 1994 and 2010 TFC criteria for diagnosis of ARVC by CMR are detailed in Figure 1.

**Figure 2:**
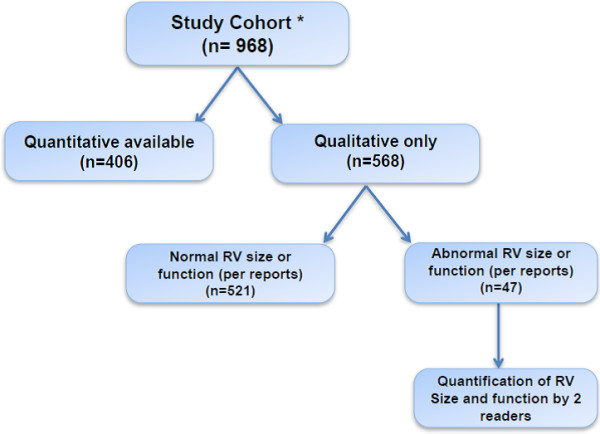
**Availability of quantitative and qualitative data.** The flow diagram shows how we did in this study when the quantitative data is not available. *All the total study cohort (n=968) had qualitative information available (segmental RV hypokinesia, dyskinesia, akinesia, aneurysm or segmental RV dilatation) per clinical reports.

CMR qualitative parameters for both 1994 and 2010 criteria (assessment of regional wall movement abnormalities and presence of RV dilation) were independently reported by experienced clinical cardiac imagers (radiologists and cardiologists with at least 5 years of experience performing and interpreting CMR). Regional wall motion abnormalities were graded as per standard department clinical procedures as 1) normal: systolic wall thickening > 40%; 2) hypokinetic: wall thickening < 40%; 3) akinetic: systolic wall thickening < 10%; 4) dyskinetic: myocardium that moved outward in systole; 5) aneurysmal: myocardial segments with persistent bulging in diastole and outward movement in systole [14].A flow diagram showed the detailed availability of quantitative and qualitative data (Figure 2). When quantitative measures of RV size and function were not available, and in whom abnormal size or function was reported, a repeat quantitative analysis by 2 qualified CMR physicians in consensus using GE Advantage Workstation with MEDIS Mass Analysis package. LV and RV end diastolic volume (LVEDV, RVEDV) and LV and RV ejection fraction (LVEF, RVEF) were calculated using a summation of disks method (Simpson’s rule) with balanced-SSFP cine images obtained in the short axis view.

For 1994 TFC, the presence of RV enlargement was determined per clinical reports when quantitative assessment was not available. Then the severity of global RV dilation and RVEF reduction was quantitatively determined according to fixed ranges from the 2010 criteria [20]. Other qualitative major and minor criteria (presence of RV aneurysms, regional RV hypokinesia, or regional RV dilatation) were identified per clinical reports of all patients.

For the 2010 TFC, the major and minor criteria were evaluated based on a combination of severe regional RV wall movement abnormalities with quantitative assessment of RV volume and function. We first determined the presence of severe regional RV wall movement abnormalities (regional RV akinesia, dyskinesia and contractile dyssynchrony) based on the CMR reports. Then, quantitative RV measures of end diastolic volume index (RVEDVI) and RV ejection fraction (RVEF) were evaluated in all patients who determined to have the presence of regional RV akinesia, dyskinesia or contractile dysynchrony, using the equipment and methods detailed above.

We utilized the 1994 and 2010 TFC for ARVC to adjudicate the CMR findings as major, minor or negative. According to the 1994 TFC, localized or generalized severe RV dilation and dysfunction with normal LV function were considered major CMR criterion for ARVC. Mild global RV dilation and dysfunction were interpreted as minor criterion.

The 2010 TFC for ARVC were applied with respect to indexed RV size (RVEDVI/BSA, ≥110 ml/m2 for males and ≥100 ml/m2 for females) or RV function (RVEF ≤ 40%) as the major CMR-based diagnostic criteria for ARVD. The milder forms of RV dilation or dysfunction (RVEDVI/BSA, ≥100 to < 110 ml/m2 for males and ≥ 90 to < 100 ml/m2 for females, RVEF > 40% to ≤ 45%) were considered as minor criteria.

In addition, all CMR exams were examined for incidental and potential alternative diagnoses. ARVC mimic diagnoses were categorized into four groups by the type of abnormality detected, including 1) cardiac displacement, 2) ischemic and/or non-ischemic cardiomyopathy, 3) myocarditis, and 4) RV pressure/volume overload. Furthermore, we recorded the presence of myocardial fat and LGE as a hypothetical CMR criterion to potentially reclassify upward both the 1994 and 2010 TFC as an additional analysis.

## Results

### Baseline characteristics

A total of 968 patients between 1995 and 2010 underwent CMR with tissue characterization for suspected ARVC. The most common symptom was palpitations (N = 250, 26%), with others including syncope (N = 124, 13%), chest pain (N = 92, 10%), and shortness of breath (N = 70, 7%). Baseline EKG showed ST-T wave abnormalities in 114 patients, right bundle branch block in 35 patients and left bundle branch block in 20 patients. The most common medications at the time of exam were beta-blockers (N = 205) and/or aspirin (N = 128). Cardiovascular risk factors were reported in 631 patients, which included hypertension (N = 156), hyperlipidemia (N = 136), and history of smoking (N = 158).

The mean age of presentation for CMR analysis was 42 ± 16 years. Overall, Quantitative measures of RV size and function were available in 406 (42%) of subjects, while qualitative data were available in the remainder 562 (58%). However, in the subjects where only qualitative data was available, abnormal RV size or function was reported in 47 patients (Figure 2). Patients had, on average, preserved LVEF (60.2 ± 7%) and RVEF (52.5 ± 6.8%). The mean RV end-diastolic volume index was 86.5 ± 23.3 cc/m^2^ (Table 1).

**Table 1:** Patient Characteristics

**Variables**	**No. of patients (n = 968)**
**Age, years**	42 ± 16
**Female, n (%)**	463 (47.8)
**LVEF, % (mean ± SD)**	60.2 ± 7.0
**RVEF, % (mean ± SD)**	52.5 ± 6.8
**RVEDVI/BSA, ml/m**^**2**^**(mean ± SD)**	86.5 ± 23.3

### 1994 and 2010 TFC CMR criteria

**Figure 3:**
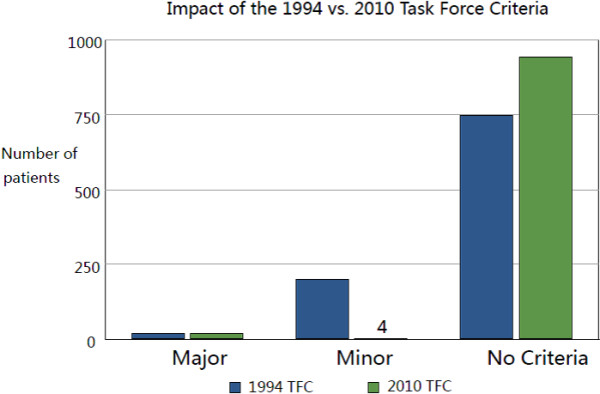
**Incidence of 1994 and 2010 ARVD criteria.** The graph shows the different incidence of patients with major criteria, minor criteria, or no criteria according to 1994 cardiac magnetic resonance criteria (blue bars) and 2010 criteria (green bars).

Proportional incidence of 1994 and 2010 TFC are reported in Figure 3.

Based on the 1994 TFC, 220 (22.7%) fulfilled either a major or a minor CMR criterion for ARVC, with 18(1.9%) fulfilling a major criterion. The majority of these patients had RV aneurysms followed by severe global RV dysfunction/dilation. A majority of the patients who fulfilled a minor criteria did so via regional RV hypokinesis (N = 133, 13.7%) (Table 2).

**Table 2:** RV function and dimensions based on 1994 original task force criteria

**Characteristic**	**All patients (N = 968)**	**% of patients**
**Major**	**18**	**1.9**
Severe global RV dilatation and Reduction of RVEF	8	0.8
RV aneurysms	12	1.2
Severe segmental RV dilatation	1	0.1
**Minor**	**202**	**20.9**
Mild global RV dilatation	34	3.5
Reduction of RVEF with normal LV	70	7.2
Regional RV hypokinesia	133	13.7
Mild segmental RV dilatation	0	0

*Abbreviations*: *RV* right ventricle, *RVEF* right ventricle ejection fraction, *LV* Left ventricle.

Applying the 2010 TFC, only 52 patients were identified presence of regional RV akinesia, dyskinesia or RV aneurysms detailed in Table 3; however, almost half of them fulfilled a major or minor criterion. Contrary to 1994 TFC, relatively fewer patients were identified as fulfilling minor criteria in 2010 modified TFC.

**Table 3:** RV function and dimensions based on 2010 revised task force criteria

**Characteristics**	**All patients (N = 968)**	**% of patients**
**Any dyskinesia, akinesia or aneurysm**	**52**	**5.4**
Dyskinesia	36	3.7
Akinesia	26	2.7
Aneurysm	12	1.2
**Major**	**21**	**21.7**
RVEDVI/BSA ≥ 110 mL/m^2^ (male) or ≥ 100 mL/m^2^ (female)	8/52	15.3
RV ejection fraction, %, <40%	21/52	40.3
**Minor**	**4**	**4.1**
RVEDVI/BSA ≥ 100 to < 110 mL/m^2^(male) or ≥ 90 to < 100 mL/m^2^ (female)	1/52	7.6

*Abbreviations*: *RV* right ventricle, *RWMA* regional wall movement abnormality, *RVEF* right ventricle ejection fraction, *RVEDV/BSA RV* end-diastolic volume index to body surface area.

Among the 220 (22.7%) patients meeting 1994 criteria for ARVC, only 25(2.6%) patients satisfied any 2010 criteria (Table 4). Among these 25 patients, 21 met a major 2010 criterion; and 4 fulfilled only minor 2010 criteria. Conversely, all patients who fulfilled a 2010 criterion for ARVC also satisfied a 1994 criterion. Eight patients that met 1994 minor criteria for ARVC were reclassified since these patients satisfied 2010 major criteria, as determined by severe reduction of RVEF and wall motion defects but without severe RV dilation or RV aneurysm. On the other hand, four patients that fulfilled a 1994 major TFC were reclassified to minor criteria or no criteria based on the 2010 TFC, which with the presence of RV aneurysm but not fulfilled 2010 quantitative criteria (Table 5). Similar results were founded when we restricting the cohort to these subjects (n = 406) with complete quantitative data available, 26.6% met at least a 1994 criterion, while only 2.9% fulfilled 2010 CMR criteria (Table 6). With inclusion of the presence of any intramyocardial fat as an additional, hypothetical criterion, 631 (65%) patients met any 1994 criteria and/or fat criteria versus 550 (57%) patients meeting any 2010 criteria and/or fat criteria (Table 7).

**Table 4:** Comparison of 1994 and 2010 MRI criteria for ARVC

***1994***	**Any TFC or fat**	**None**	**Total**
***2010***			
**Any TFC or fat**	25	0	25
**None**	195	748	943
**Total**	220	748	968

**Table 5:** Comparison of 1994 and 2010 MRI major and minor criteria for ARVC

***1994***	**Major**	**Minor**	**None**	**Total**
***2010***				
**Major**	13	8	0	21
**Minor**	1	3	0	4
**None**	4	191	748	943
**Total**	18	202	748	968

**Table 6:** Comparison of 1994 and 2010 MRI criteria for ARVC in the quantitative cohort only

***1994***	**Major or minor**	**None**	**Total**
***2010***			
**Major or minor**	12	0	12
**None**	96	298	394
**Total**	108	298	406

**Table 7:** Comparison of 1994 and 2010 MRI criteria plus fat for ARVC

***1994***	**Any TFC or fat**	**None**	**Total**
***2010***			
**Any TFC or fat**	25	0	25
**None**	195	748	943
**Total**	220	748	968

Among these 220 patients with any CMR 1994 or 2010 criteria, 68 (30.9%) had CMR evidence of LV impairment. Within this subgroup, 6 (2.7%) patients had LV wall motion abnormality, LV dilatation in 26 (11.8%), and reduced LVEF in 50 (22.7%) of these patients. 2(0.9%) had apical aneurysms.

### Potential ARVC mimics and other incidental cardiac findings

We also examined the presence of other common cardiac and extra-cardiac findings that could mimic ARVC (Table 8). Of interest, 16 patients were reported to have signs of RV pressure or volume overload, without further explanation. Twelve patients had cardiomyopathy (ischemic or non-ischemic). Other mimics included cardiac displacement (N = 8), tricuspid regurgitation (N = 8), myocarditis (N = 7), cardiac sarcoidosis (N = 2), and kyphoscoliosis. Other unexpected diagnoses that were not directly linked to ARVC but were detected incidentally included congenital heart diseases (N = 7), regurgitant valvular diseases (N = 6), RV lipomatosis (N = 7), coronary anomalies, and LV hypertrophy (Table 9). In total, 89 (9.2%) patients were found to have an alternative diagnosis rather than ARVC, 43 (4.4%) of which could mimic ARVC.

**Table 8:** ARVC mimics

**ARVD mimics**	**All patients (N = 968)**
**Cardiac displacement**	8
PectusExcavatum	6
Kypohoscoliosis	1
Narrow A-P chest diameter	1
**Ischemic and non-ischemic cardiomyopathy**	12
RV inferior infarction	1
Amylodosis	1
Tachycardia mediated cardiomyopathy	1
LVNC	2
Dilated cardiomyopathy	1
Sarcoidosis	2
Hypertrophy cardiomyopathy	4
**Cardiac inflammatory or infectious disease**	7
Myocarditis	7
**RV pressure/volume overload disease**	16
Pulmonary hypertension	1
Sinus venosus ASD	4
Pulmonic regurgitation	5
Tricuspid regurgitation	8
**Total**	**43(4.4%)**

*Abbreviations:A-P* anterior-posterior, *RV* right ventricle, *LVNC* left ventricular non-compaction, *ASD* atrial septal defect.

**Table 9:** Other diagnoses and findings from suspected ARVC patients

**Other unexpected diagnoses and findings**	***N***
**Congenital heart disease**	7
Left sided SVC	1
Bicuspid aortic valve	2
Anomalous right coronary artery	3
Anomalous pulmonary venous	1
**Ischemic cardiomyopathy**	3
LV infarction	2
LV aneurysm	1
**Cardiac inflammatory or infectious disease**	2
Constrictive pericarditis	1
Acute pericarditis	1
**Valvular disease**	6
Mitral regurgitation	2
Aortic regurgitation	4
**Non specified finding**	11
Pericardial effusion	7
Pleural effusion	4
**Cardiac tumors**	7
Right ventricular lipomatosis	6
InteratrialMass	1
**Abnormal, but non-pathologic conditions**	10
LV hypertrophy	3
Prominent trabeculations	1
Trace pulmonary insufficiency	3
RV hypertrophy	3
**Total**	**46(4.7%)**

*Abbreviations*: *SVC* superior vena cava, *RV* right ventricle, *LV* left ventricle.

### Late gadolinium enhancement and ARVC criteria and mimic

**Figure 4:**
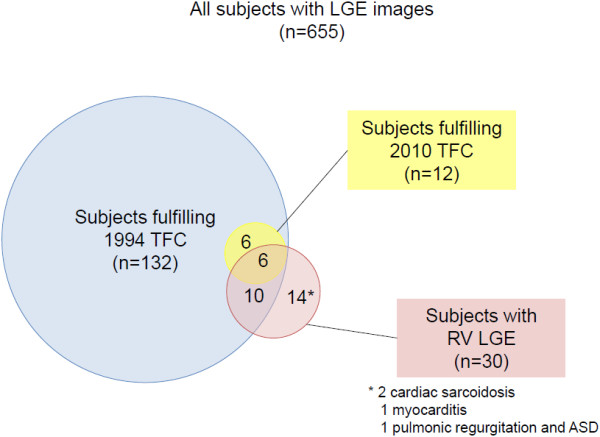
**Relationships between 1994 and 2010 TFC with LGE.** The white box represents all patients with LGE acquired. The blue circle represents subjects fulfilling 1994 CMR task force criteria. The yellow circle represents subjects fulfilling the 2010 CMR task force criteria. The red circle shows subjects with the presence of RV late gadolinium enhancement.

655 subjects underwent LGE imaging, and among these patients, 132 (20.1%) fulfilled at least a 1994 TFC, while only 12 (1.8%) met a 2010 TFC. Furthermore, 30 (4.6%) had positive findings of LGE in the RV, 15 (2.2%) had LV LGE, and 7 (1.1%) of these patients with both RV and LV LGE. Among subjects with any 1994 TFC, 16 out of 132 patients were positive for RV LGE. In the patients with any 2010 TFC, 6 out of 12 patients had RV LGE (Figure 4). Of the remaining 14 patients with RV LGE not fulfilling any TFC, two patients were diagnosed with cardiac sarcoidosis (positive for both RV and LV LGE), one patient had a final clinical diagnosis myocarditis, one patients had ischemic RV infarction, and one had pulmonic regurgitation and prior ASD; the remaining 9 patients did not have any specific MRI diagnosis but did have RV LGE. 7 patients with LV LGE also had RV LGE; all of these met at least one TFC. The other 8 patients with positive LV LGE all had a final diagnosis which could mimic ARVC.

## Discussion and conclusion

Although ARVC is a rather rare disorder, is a frequent reason for referral to CMR. In this light, many radiologists and cardiologists who interpret CMR have become quite familiar with this relatively uncommon disease entity. According to the 2010 Task Force Criteria, meeting even a single major criterion (which can be CMR based) allows for a *possible* diagnosis of ARVC (the final diagnosis of which requires further clinical input). Therefore, there is a significant burden upon cardiac imagers to provide an accurate assessment and documentation of CMR findings, as this imaging test may have important repercussions for clinical management, including the indication for an implantable cardiac defibrillator placement.

In our study of almost one thousand adult patients referred for CMR during a 15-year period at a tertiary academic referral center with the primary indication of evaluation for ARVC, we report several significant findings. First, the vast majority of patients (943 out of 968, or 97.4%) undergoing cardiac MRI do not meet any criteria for ARVC according to the 2010 Task Force Criteria. This greater selectivity is related to the introduction of more objective and quantitative parameters for diagnosis with the 2010 criteria as compared to the 1994 criteria. The low positivity rate of CMR for ARVC could be considered intuitive given the rarity of the disease, and may possibly reflect a low threshold for ordering a CMR to rule out this entity. This finding becomes important for lower volume centers to recognize, especially if disease prevalence at their centers is similarly low or even lower than at our tertiary referral center.

In addition, we have shown that there is significant re-classification of patients comparing the use of the 1994 versus the 2010 Task Force Criteria. The greatest shift in classification occurred in those patients who met the minor criteria according the 1994 Task Force Criteria. Interestingly, the vast majority of these were re-classified as having no criteria according to the 2010 criteria, yet 8 patients were upgraded to meeting major criteria. These findings suggest that the 2010 criteria are “stricter” by nearly eliminating the category of patients satisfying only minor criteria. This near dichotomization of cardiac MRI categorization (into major and no criteria) suggests a potentially increased importance for CMR in making the diagnosis of ARVC, as meeting the CMR major criterion allows for a possible diagnosis of ARVC (imaging findings alone are insufficient for diagnosis). Conversely, meeting no CMR criteria will eliminate any imaging criteria contribution to the overall clinical diagnosis of ARVC, so that significant non-imaging criteria must therefore be present to establish the diagnosis.

Our findings reinforce and confirm the predictable results of Vermes *et al*’ s study by showing an overall reduction in the prevalence of any positive CMR criteria with the revised 2010 Task Force guidelines [15,19]. However, our study included a much larger sample size (968 versus 294 patients), and we included *all consecutive* patients referred with an indication of evaluating for ARVC whereas Vermes *et al*. only evaluated patients referred by electrophysiologists. Therefore, our findings may be more applicable to a community practice population (the nature of our referral base made complete electrophysiology records unavailable in a number of externally referred patients). A key difference is that although the number of patients with any positive CMR criteria was substantially reduced with the 2010 criteria, the number of patients with major criteria was relatively similar using both TFC classification schemes. Vermes *et al*. stated that their relatively large number of patients meeting major criteria according to the 1994 criteria was due to a local tendency to overcall “microaneurysms”, however this terminology was apparently not described in the 1994 criteria (only aneurysms are discussed). Our readers tended to use descriptive terminology (e.g. localized bulge throughout systole and diastole), in addition to the keyword such as “aneurysm”. In addition, aneurysms represented a minority of the regional wall motion abnormalities that were detected on the examined studies.

Aside from these findings, we have taken a step further by evaluating two other items of practical importance: (1) assessing for ARVC “mimics” and other diagnostic findings, (2) evaluating the value of intra-cardiac fat for CMR diagnosis of ARVC. Nearly 10% of the patients in our cohort had either potential ARVC mimics or other clinically important cardiac diagnoses. Although the importance of dedicated CMR sequences to distinguish between ARVC and cardiac sarcoidosis and myocarditis have previously been described [21-24], we found many patients with other cardiomyopathies, as well as RV volume and pressure overload conditions (atrial septal defect, tricuspid regurgitation, etc.) that may explain a patients’ clinical presentation that led to a CMR investigation for ARVC criteria.

Furthermore, much emphasis has historically been placed on fat assessment of the right ventricular myocardium by CMR, with the inclusion of dedicated fat suppression sequences for this purpose at many institutions including our own. Traditionally, the conventional wisdom had been that MRI characterization of fat could serve as a “pathologic equivalent” and thus meet the MRI equivalent of the major 1994 TFC (which required pathologic tissue confirmation of fat). Therefore, we analyzed whether adding the presence of fat in the RV myocardium as a CMR criterion would provide any untility in our patient population. As expected, we found that many patients have some degree of RV myocardial fat, and that there were no patients not fulfilling 1994 criteria whom would have met 2010 criteria (had RV myocardial fat been hypothetically included as a criterion). These findings suggest a limited utility for dedicated, time-intensive sequences for fat evaluation in patients undergoing CMR evaluation for ARVC.

Lastly, abnormal late gadolinium enhancement imaging abnormal in contrast-enhanced MRI can be detected in the right and left ventricular myocardium in patients with suspected arrhythmogenic right ventricular cardiomyopathy suggesting areas of intramyocardial fibrous tissue [25]. In our study, we found that less than half of the patients with positive RV LGE also met either the 1994 or 2010 CMR criteria. All the patients with positive LV LGE but without CMR criteria were ultimately diagnosed with ARVC mimics. These findings suggest that late gadolinium enhancement play a very important role in evaluation of ARVC and is also helpful to identify ARVC Mimics.

LV involvement of arrhythmogenic right ventricular has been identified as more common finding in patients with ARVC. Sen-Chowdhry *et al.* reported among 200 affected patients, 76.3% patients were found with CMR evidence of LV involvement [26]. In our study, we found 30.9% of the patients with any 1994 or 2010 CMR criteria also had an LV size or function abnormality.

There are, however, several limitations of our retrospective, imaging-based, study. First, we did not have complete clinical information for all patients in order to fully assess clinical Task Force Criteria beyond CMR (e.g. signal-averaged ECG, Holter monitor, etc.), and therefore, we did not have a complete “gold standard” for determining a probable or definitive diagnosis of ARVC. This was secondary to a wide referral base in which many patients only came to our center only for CMR, and therefore did not receive further clinical follow-up at our institution. Second, we pooled cases over many years with varying image quality, during which time technical pulse sequences improved, and clinical readers gained experience. The segmentation of the right ventricle to calculate volumetric parameters was initially not standardized and in some older cases was not performed. Manual RV tracing for quantization is known to be time consuming and to be operator-dependent [27]. However, our intention was to include *all* cases across this time period (which fell between the 1994 and 2010 TFC, thus eliminating potential bias due to knowledge of the 2010 TFC) to make our results more practically applicable. In addition, in our analysis of ARVC mimics and other cardiac diagnoses, we did not systematically assess whether CMR was the first or only test to detect these diagnoses, as some of these patients may have had additional testing performed at other institutions that was not accessible to us.

Considerations that could be incorporated into future guideline documents for CMR imaging of ARVC include whether other quantitative methods for right ventricular assessment (e.g. tricuspid annular systolic plane excursion [TAPSE], time-volume curves [27], phase-contrast MRI [28], or perhaps myocardial strain analysis) may be useful alternatives to traditional quantification of RV volumes and functional parameters [29]. Also, long-term clinical outcome and follow-up data such as defibrillator implantation and appropriate shock therapies, ventricular arrhythmias, and sudden cardiac death are needed for these patients to answer questions regarding the efficacy of current Task Force Criteria in directing further management.

A minority (2.6%) of clinically suspected ARVC patients undergoing CMR met the 2010 TFC at our center. While this suggests a low diagnostic yield of CMR for ARVC (and we also noted no additional benefit of fat assessment), we demonstrated a significantly higher yield for potential ARVC mimics (cardiomyopathies, RV volume and pressure overload conditions) at 4.4% [30,31]. Although our results are provocative, a larger, multicenter registry of CMR for ARVC would be preferable to confirm our single-center results.

In summary, application of the 2010 TFC resulted in a reduction of total patients meeting any diagnostic MRI criteria for ARVC from 22.7% to 2.6% versus application of the 1994 TFC. The hypothetical inclusion of myocardial fat as a criterion would not have changed the TFC diagnosis in any patients. CMR identified alternative cardiac diagnoses in 9.1% of patients, and 4.4% of all diagnoses were potential mimics of ARVC.

## Abbreviations

TFC: Task force criteria; CMR: Cardiovascular magnetic resonance; ARVC: Arrhythmogenic right ventricular cardiomyopathy; RV: Right ventricle; RVEDV: Right ventricle end diastolic volume; RVEF: Right ventricle ejection fraction; LVEDV: Left ventricle end diastolic volume; LVEF: Left ventricle; BSA: Body surface area; A-P: Anterior-posterior; LVNC: Left ventricular non-compaction; ASD: Atrial septal defect; SVC: Superior vena cava.

## Competing interests

The authors declare that they have no competing interests.

## Authors’ contributions

TL and AP were involved in designing the study and also collecting and analyzing the data, drafting and submitting the manuscript. UCS was involved in designing the study, reviewing cases and drafting the manuscript. YV, DV PD ML AS MJ HL MS HM were involved in collecting the data and revising the manuscript. AML and SD were involved in revising the manuscript. SA, GH UH was involved in overseeing the study. BBG was involved in designing the study, reviewing cases and drafting the manuscript. All authors have read and approved the final manuscript.
